# The Clinical Application of the ARi^®^ Implant System in Severely Resorbed Anterior Alveolar Ridges: A Case Report

**DOI:** 10.3390/dj13060241

**Published:** 2025-05-28

**Authors:** Kwang-Bum Park, Hyun-Wook An, Keun-Oh Park, Min-Ho Hong

**Affiliations:** 1 MegaGen Implant Co., Ltd., Daegu 42921, Republic of Korea; 2 Department of Dental Laboratory Science, College of Health Sciences, Catholic University of Pusan, 57, Oryundae-ro, Geumjeong-gu, Busan 46252, Republic of Korea

**Keywords:** implants, alveolar resorption, alveolar ridge, dental esthetics

## Abstract

**Background/Objectives**: The rehabilitation of severely resorbed anterior alveolar ridges presents significant clinical challenges due to esthetic demands and the limited bone volume in this region. Basal cortical implants, which are designed to engage dense basal bone, could offer an alternative by providing stable anchorage in compromised sites. **Methods**: This report evaluates the ARi^®^ Implant System, which features cortical anchorage and a calcium-incorporated nanostructured surface (XPEED^®^) in two anterior ridge defect cases. Soft tissue augmentation using a vascularized interpositional periosteal (VIP) flap was applied in one case, and biphasic calcium phosphate (BCP) grafting and collagen membranes were employed for ridge contouring in both cases. **Results**: At a two-year follow-up, both cases showed stable peri-implant tissues and satisfactory esthetic results. **Conclusions**: Although basal cortical implants provide good primary stability, their use does not eliminate the need for bone augmentation, especially in the anterior esthetic region. Future clinical studies are required to substantiate long-term outcomes and broader applicability.

## 1. Introduction

Esthetics and functionality are critical aspects of dental treatment, particularly in the anterior maxillary region, due to its prominent visibility and influence on patient self-esteem and social interactions [1]. Tooth loss in this region can significantly impact psychological well-being, emphasizing the importance of meticulous treatment planning and technical precision to satisfy high esthetic expectations compared to posterior restorations [2]. Consequently, restoring anterior teeth presents unique challenges and requires careful consideration of esthetic harmony and functional recovery [3].

Implant placement has become a well-established and predictable method for rehabilitating missing teeth. However, achieving successful implant outcomes necessitates sufficient alveolar bone volume, defined as at least 13–15 mm in length and 5–7 mm in width [4]. In cases where these criteria are unmet due to severe alveolar ridge resorption, additional surgical procedures such as sinus augmentation, extensive bone grafting, and nerve lateralization may be indicated [5]. Such advanced surgical interventions add complexity to the treatment process and increase patient morbidity, cost, and the overall duration of rehabilitation, potentially limiting accessibility to effective care.

As a result, conventional implant treatments frequently present clinical challenges, particularly in scenarios involving significant bone atrophy and compromised soft tissue conditions. The need for extensive bone grafting and guided bone regeneration (GBR) procedures in these situations increases the risk of complications, prolongs treatment timelines, and contributes to patient discomfort [5,6,7]. It is important to emphasize that, especially in the esthetic zone, the need for bone regeneration is predominantly influenced by the degree of alveolar ridge resorption and the associated anatomical constraints, rather than solely depending on the type of implant system utilized [6,7,8]. Thus, even advanced implant systems designed to enhance primary stability and minimize bone augmentation cannot completely eliminate the requirement for additional bone grafting procedures in cases of severely compromised ridge conditions. Studies have emphasized that GBR techniques for vertical ridge augmentation generally require prolonged healing, often exceeding six months before implant placement; this further complicates the clinical management of severely resorbed alveolar ridges [8,9].

Basal cortical implants, which are specifically designed to achieve stable anchorage in dense basal cortical bone rather than relying on alveolar cancellous bone, were introduced to overcome these challenges [10]. Basal cortical bone possesses inherent structural resilience, higher mineral density, and resistance to resorption, providing robust and predictable implant anchorage even under severely compromised alveolar ridge conditions [11]. The basal cortical implant (BCI) system has evolved substantially over several decades, leading to significant enhancements in design and functionality. The first single-piece basal implant was introduced by Dr. Jean-Marc Julliet in 1972 [12], followed by the development of the disk implant system by Dr. Gerard Scortecci in the 1980s [12]. Subsequently, advancements made by Dr. Stefan Ihde further refined basal osseointegrated implants (BOIs), optimizing their ability to efficiently transmit occlusal forces through cortical bone and reduce marginal bone stress [12].

**Figure 1:**
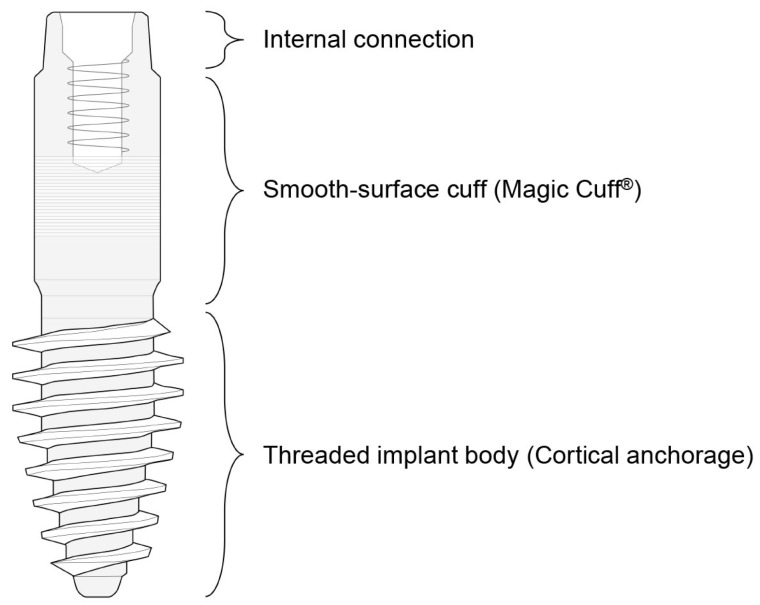
Overall structure of the ARi^®^ Implant. The implant comprises three main components: a threaded body at the lower portion, a cuff in the middle, and a connection at the upper portion. The cuff features a micro-groove designed to facilitate soft tissue stability and integration.

The ARi^®^ Implant System has emerged as an effective basal cortical implant designed to address severe ridge atrophy in esthetically critical regions such as the anterior maxilla and mandible (Figure 1).

The overall structure of the ARi^®^ Implant System consists of two primary components: a threaded implant body, which achieves anchorage in basal cortical bone, and the Magic Cuff^®^, which facilitates abutment connection. The implant body features sharp, curved threads designed to enhance mechanical stability by achieving anchorage in basal cortical bone. This thread design maximizes initial fixation strength upon insertion, which may help reduce the extent of bone augmentation procedures required in selected patients, although it does not completely eliminate the need for grafting, especially in cases of severe ridge resorption. The ARi^®^ Implant’s surface incorporates a nanostructured, calcium-incorporating surface treatment known as XPEED^®^ [13,14,15]. This advanced coating significantly enhances early osteoblastic activity, accelerates apatite formation, and promotes rapid bone-to-implant bonding. XPEED^®^ surfaces show superior biological responses, including improved osteoblast proliferation, viability, and differentiation, ultimately resulting in enhanced bone-to-implant contact (BIC) and greater implant stability. Moreover, implants treated with the XPEED^®^ coating exhibit higher removal torque values, indicative of superior mechanical integration with the surrounding bone, and promote more homogeneous and densely mineralized bone deposition [13,14]. The ARi^®^ Implant also includes the Magic Cuff^®^, an intermediary connection designed to ensure stable and reliable soft tissue support while minimizing pressure on the alveolar ridge. This slender, cylindrical connection is characterized by a smooth surface analogous to the machined collar of gingival-level implants to enhance peri-implant soft tissue stability and minimize mucosal complications. Additionally, while this study primarily emphasizes the applicability of basal cortical implants in esthetically demanding anterior regions, basal cortical implants could also be beneficial in posterior regions due to their cortical anchorage properties. Specifically, short implants can often be successfully employed in posterior regions with limited alveolar bone height, potentially reducing the need for complex regenerative procedures. Previous randomized controlled trials (RCTs) have demonstrated favorable outcomes with short implants in posterior regions, supporting their viability as a minimally invasive alternative [16,17].

Despite these advantageous structural and biological characteristics, clinical case reports detailing the practical efficacy and clinical outcomes of the ARi^®^ Implant System remain limited. This scarcity of documented clinical cases underscores the need for further research to validate and substantiate the clinical effectiveness of the ARi^®^ Implant in various complex clinical scenarios. Therefore, this study aims to evaluate the applicability and efficacy of basal cortical implants through a case report involving restoration in the anterior region. Specifically, it explores the potential applicability of the ARi^®^ Implant System in thin ridge cases, discussing possible clinical advantages and limitations compared to conventional methods. These findings may provide insights to support clinical decision-making in anterior restorations.

## 2. Case Reports

### 2.1. Case Report 1

**Figure 2:**
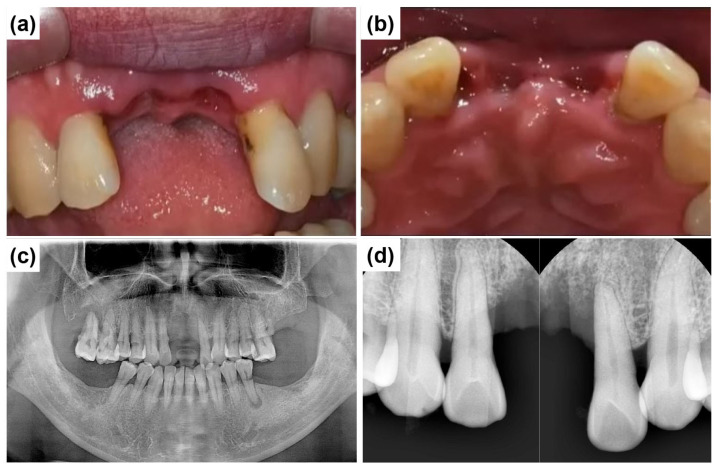
Preoperative clinical and radiographic evaluation. (**a**) Labial intraoral view of the anterior edentulous region (at the initial visit) shows the absence of maxillary central incisors (#11 and #21) and significant alveolar ridge resorption. (**b**) Occlusal intraoral view of the maxillary anterior ridge, severe horizontal ridge atrophy. (**c**) Initial panoramic radiograph shows the overall periodontal condition and alveolar bone status. (**d**) Preoperative intraoral periapical radiograph of the maxillary anterior region illustrates alveolar bone loss and root morphology before implant placement.

A 58-year-old male patient reported to Mir Dental Hospital at Daegu with missing maxillary central incisors (teeth #11 and #21). The patient was a heavy smoker but had no significant systemic conditions. He revealed that he had lost the teeth three months prior due to periodontal disease. Intraoral examination confirmed the absence of teeth #11 and #21. Figure 2a,b illustrate the clinical presentation at the initial visit, demonstrating severe alveolar ridge resorption and distinct U-shaped gingival recession. Radiographic evaluations, including cone-beam computed tomography (CBCT) and clinical examinations, were conducted to assess bone loss and optimize treatment planning. A panoramic radiograph revealed generalized poor periodontal health (Figure 2c), with notable horizontal alveolar bone loss and vertical bone defects in the edentulous region. Significant labial alveolar bone loss was observed, especially mesially around tooth #22 (Figure 2d).

A detailed periodontal examination further revealed that tooth #22 exhibited severe periodontal compromise, including probing depths ranging from 7 to 9 mm, clinical attachment loss exceeding 10 mm, Grade 3 mobility, bleeding on probing (BOP), and notable gingival recession of 5 mm. Given these periodontal parameters, the tooth had an unfavorable prognosis and warranted extraction.

The patient requested implant placement for the immediate restoration of missing teeth. Various treatment options were discussed to optimize esthetics and function. It was decided that an endosteal implant would be placed at the site of tooth #11. Extraction of tooth #22 was planned due to significant alveolar bone resorption and periodontal compromise, followed by the placement of an ARi^®^ Implant to achieve stable cortical bone anchorage.

**Figure 3:**
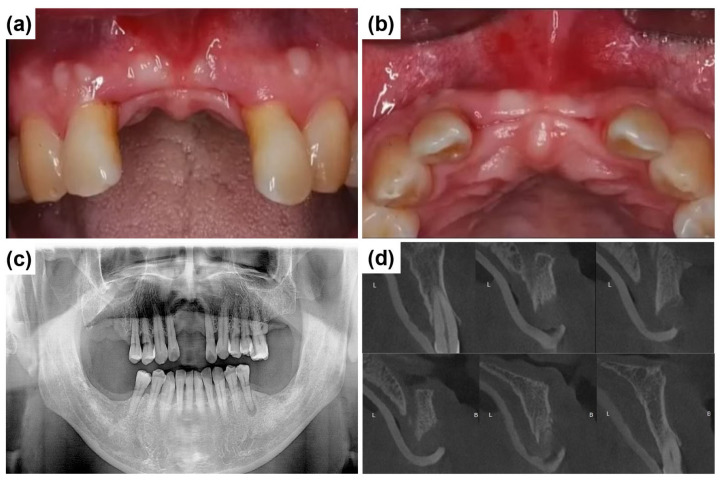
Preoperative clinical and radiographic evaluation three months after the initial visit. (**a**) The labial intraoral view shows the anterior edentulous ridge with significant alveolar ridge resorption. (**b**) The occlusal intraoral view shows horizontal ridge atrophy. (**c**) The panoramic radiograph, taken three months post initial examination, indicates overall periodontal condition and alveolar bone status before implant placement. (**d**) Cone-beam computed tomography (CBCT) images illustrate progressive thinning of the anterior alveolar ridge, emphasizing the extent of ridge resorption and its potential impact on implant placement. B and L indicate buccal and lingual directions, respectively.

Figure 3a,b show the edentulous anterior region on the day of implant placement, three months after the initial visit, illustrating significant soft tissue healing due to improved hygiene care. Compared to Figure 2a,b, the gingival condition has improved. Although implant placement was not challenging, the primary concern was managing papillary recession and ridge depression between the central incisors. Figure 3c shows a panoramic radiograph taken three months after establishing the treatment plan. During this period, multiple posterior teeth (#16, #17, #26, #27, #36, #37, #46, and #47) were extracted due to generalized advanced periodontal disease and poor prognosis. These teeth exhibited generalized advanced periodontal disease, with extensive clinical attachment loss, severe mobility (Grades 2 and 3), deep periodontal pockets (≥7 mm probing depths), and radiographic evidence of advanced alveolar bone loss, rendering them prognostically hopeless. Therefore, extraction was the most clinically appropriate and standard treatment decision. However, this case report focuses exclusively on anterior implant treatment. Figure 3d illustrates the notably thin anterior alveolar ridge. An ARi^®^ Implant was selected for tooth #22 to maximize cortical bone anchorage and minimize implant exposure risk.

**Figure 4:**
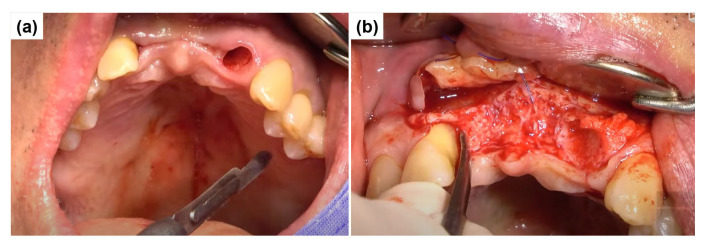
Surgical procedure for implant placement and VIP flap preparation. (**a**) The socket of tooth #22 after extraction shows extensive mesial alveolar bone loss with significant cortical bone exposure. (**b**) The initial incision for the VIP flap begins from the maxillary left second premolar. The incision line is placed approximately 3 mm apical to the marginal gingiva with a depth of about 1 mm. An additional incision is extended palatally in the anterior pontic region to facilitate adequate flap mobilization.

Before surgery, tooth #22 was strategically extracted (Figure 4a). The surgical approach for implant placement comprised bone grafting and the vascularized interpositional periosteal (VIP) flap technique. After preoperative scaling, the patient received antibiotic prophylaxis with 500 mg of amoxicillin sodium (Augmentin; Ilsung Pharmaceutical, Seoul, Republic of Korea) three times daily for seven days, beginning one day before surgery.

Local anesthesia was administered with 2% lidocaine containing 1:100,000 epinephrine (Yuhan, Seoul, Republic of Korea). The infraorbital nerve and superior alveolar nerve were anesthetized. Soft tissue management was performed simultaneously at sites #11 and #22. After extracting tooth #22, the VIP flap procedure was initiated with an incision starting from the maxillary left second premolar. The incision line was placed approximately 3 mm from the marginal gingiva at a depth of about 1 mm. Additionally, an incision was extended palatally in the anterior pontic region. Clinical examination of the extraction socket at #22 revealed extensive mesial bone loss, exposing a significant portion of the underlying bone (Figure 4b).

**Figure 5:**
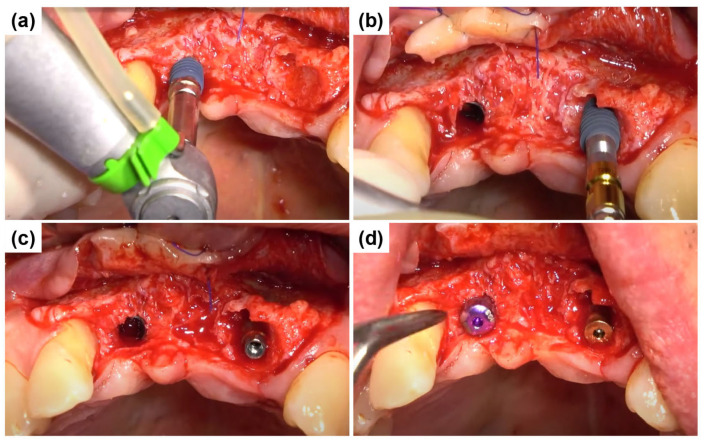
Implant placement and primary closure. (**a**) Conventional endosteal implant placement (Blue Diamond implant) in the fully healed extraction socket of tooth #11 using a standard protocol. (**b**) Placement of an ARi^®^ Implant in tooth #22 to compensate for substantial mesial and distal bone level discrepancies and a thin labial bone plate, enabling enhanced socket remodeling. (**c**) Final positioning of a 4.1 × 10.0 mm implant in tooth #11 and a 4.0 × 9.0 mm implant in tooth #22, achieving an initial fixation torque of 40 N/cm^2^. (**d**) Primary closure was obtained to facilitate optimal healing and soft tissue adaptation.

The central incisor (#11) had a fully healed extraction socket, allowing for conventional endosteal implant placement using a standard protocol (Figure 5a). In contrast, tooth #22 exhibited a substantial discrepancy between mesial and distal bone levels with a thin labial bone plate. The ARi^®^ Implant System was selected for placement to accommodate potential socket remodeling and enhance primary stability (Figure 5b). The ARi^®^ Implant System effectively addresses these concerns using basal cortical bone for enhanced anchorage. A 4.1 × 10.0 mm implant (Blue Diamond implant, MEGAGEN Implant Co., Ltd., Daegu, Republic of Korea) was placed for tooth #11. A 4.0 × 9.0 mm implant (ARi^®^ Implant, MEGAGEN Implant Co., Ltd., Daegu, Republic of Korea) was inserted for tooth #22 (Figure 5c). Both implants exhibited satisfactory primary stability, with an initial fixation torque of 40 N/cm^2^ at the time of placement. Primary closure was achieved in the subsequent phase to optimize healing and soft tissue contour (Figure 5d).

**Figure 6:**
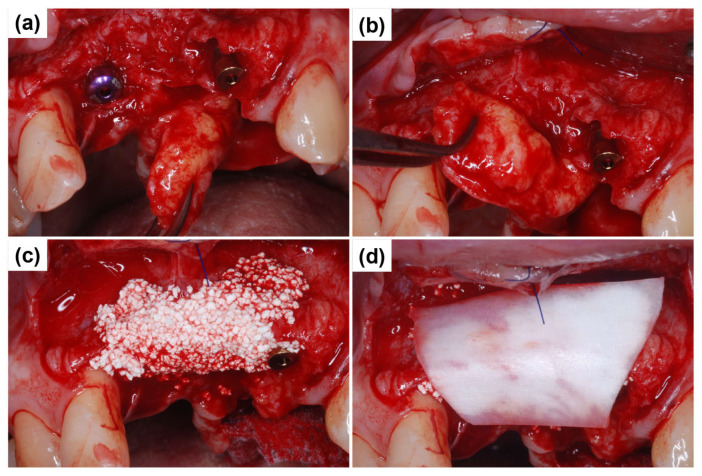
VIP flap procedure and bone contour augmentation for ridge regeneration. (**a**) Vascularized interpositional periosteal (VIP) flap, demonstrating its rotational range for optimal soft tissue coverage. (**b**) Assessment of flap mobility to ensure adequate positioning and fixation for ridge augmentation. (**c**) Application of a biphasic calcium phosphate (BCP) graft to reconstruct the ridge crest and enhance bone volume. (**d**) Lyoplant collagen membrane placement over the grafted site to prevent particle migration and facilitate stable bone regeneration.

Next, the primary objective was to restore the overall ridge contour. As the remaining anterior soft tissue alone was inadequate for additional ridge augmentation, a vascularized interpositional periosteal (VIP) flap was used by rotating the palatal connective tissue. The VIP flap technique is particularly beneficial in cases requiring effective soft tissue augmentation. Figure 6a illustrates the flap design during the VIP flap procedure. The rotational range of the flap was assessed to ensure adequate mobility for proper positioning and fixation. Bone Matrix I grafting was incorporated to enhance the volumetric stability of the ridge, allowing for secure flap positioning with sufficient mobility (Figure 6b). A healing abutment measuring 3 mm in height was connected to the Blue Diamond implant placed at #11, functioning as a vertical soft tissue scaffold to support gingival contour during healing. Next, a biphasic calcium phosphate (BCP) graft (Bone Matrix I, MEGAGEN Implant Co., Ltd., Daegu, Republic of Korea) was applied to establish a smooth ridge crest (Figure 6c). A Lyoplant collagen membrane (Lyoplant, Aesculap AG, Tuttlingen, Germany) was carefully positioned over the grafted area to prevent graft particle displacement and promote stabilization (Figure 6d).

**Figure 7:**
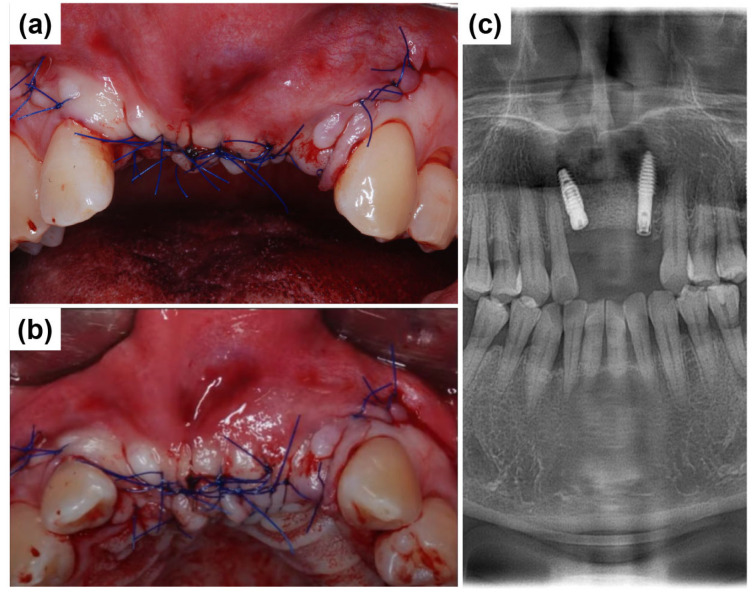
Primary closure and postoperative radiographic assessment. (**a**) Labial intraoral view showing flap repositioning and primary closure using 4-0 Vicryl sutures following implant placement. (**b**) Occlusal intraoral view. (**c**) Postoperative panoramic radiograph depicting the final placement of implants in the anterior maxillary region.

After securing the membrane and placing the bone graft, the flap was repositioned passively to avoid tension. Primary closure was achieved using 4-0 Vicryl sutures (Dafilon; B. Braun Melsungen AG, Melsungen, Germany). Simple interrupted sutures were placed along the incision line on the alveolar ridge and at the interdental papillae adjacent to the surgical site (Figure 7a,b). Figure 7c presents a postoperative radiograph of the anterior region following implant placement, demonstrating the final positioning of the implants. Long-term stability and successful osseointegration can be expected with favorable wound healing.

**Figure 8:**
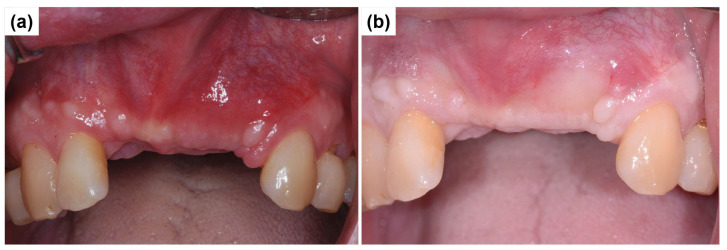
Soft tissue healing progression following implant surgery. (**a**) Intraoral clinical view showing soft tissue healing three weeks postoperatively, demonstrating progressive tissue adaptation. (**b**) Intraoral view at three months postoperatively, illustrating well-stabilized peri-implant soft tissues with improved ridge contour.

Figure 8a illustrates the soft tissue healing progression three weeks postoperatively. Compared to the mesial aspect of the #23 canine, which maintained healthy proximal tissue, the mesial proximal tissue of the #12 lateral incisor exhibited recession, probably due to prior root contamination from periodontitis. However, the vertical depression in the central incisor region showed successful recovery, resulting in a smooth gingival contour. Figure 8b presents intraoral clinical and radiographic images obtained three months after implant surgery, demonstrating well-stabilized soft tissue healing. The images indicate progressive adaptation of peri-implant tissues, suggesting favorable healing outcomes at three months postoperatively.

**Figure 9:**
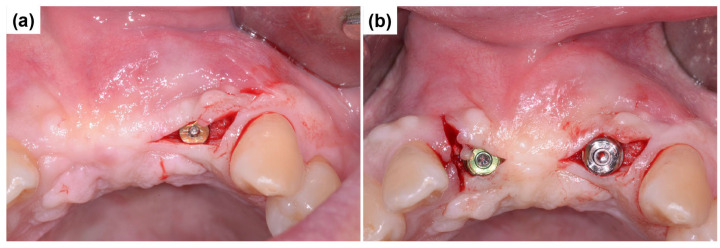
Three-corner flap formation and placement of scannable healing abutments during secondary surgery. (**a**) Formation of a three-corner flap to provide adequate access while preserving peri-implant soft tissue during secondary surgery. (**b**) Following flap elevation, scannable healing abutments were placed to promote soft tissue healing and enable precise digital impression-taking for prosthetic rehabilitation.

During the secondary surgery, a three-corner flap was carefully formed (Figure 9a) to provide adequate access to the implant sites while preserving peri-implant soft tissues. Following flap elevation, scannable healing abutments were placed (Figure 9b) to promote optimal soft tissue healing and enable precise digital impression-taking for subsequent prosthetic rehabilitation.

**Figure 10:**
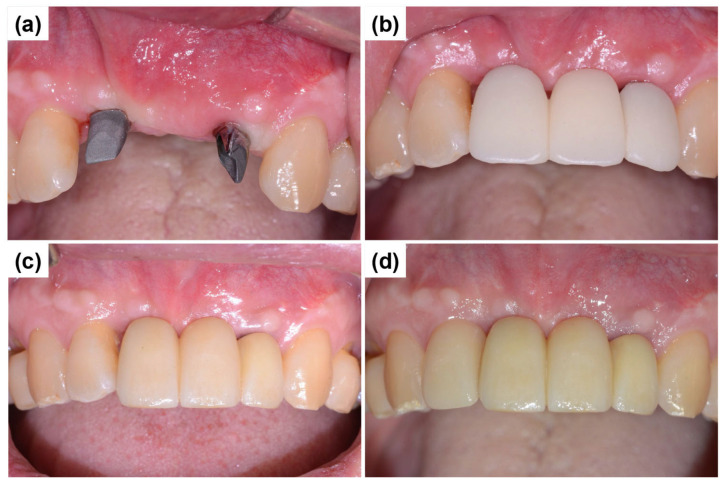
Connection of customized abutments, provisional prosthesis placement, and final restoration. (**a**) Fabrication and connection of customized abutments one month after the secondary surgery, following the assessment of gingival healing. (**b**) Placement of a PMMA temporary bridge using temporary cement on the customized abutments to facilitate continued soft tissue adaptation before final prosthesis placement. (**c**) Final prosthesis placement. (**d**) The final esthetic outcome after laminate treatment shows improved anterior esthetics and overall functional integration.

One month after the secondary surgery, the gingival healing status was evaluated, and customized abutments were fabricated and connected to the implants (Figure 10a). Subsequently, a PMMA (polymethylmethacrylate) temporary crown was fabricated, and the provisional prosthesis was attached to the customized abutments using temporary cement (Figure 10b). Figure 10c shows the final prosthesis placement following the removal of the provisional restoration. Esthetic concerns were observed, specifically mesial papillary recession at the right lateral incisor, resulting in compromised gingival harmony and papillary contour. Following comprehensive discussions with the patient regarding potential esthetic improvements, a laminate veneer restoration was planned and executed to address the patient’s explicit esthetic concerns. Although laminate veneer placement may not be considered an essential procedure from a strictly periodontal standpoint, it was justified by the patient’s esthetic demands, improved self-confidence, and overall satisfaction with the final restoration (Figure 10d). Although the crown length of the left lateral incisor appeared slightly shorter, the patient expressed satisfaction with the overall outcome, and a follow-up plan was established. Despite initial concerns regarding gingival contour irregularities and alveolar bone deficiencies, a functionally and esthetically stable outcome was achieved, demonstrating the efficacy of the applied treatment approach. The decision to implement a three-unit bridge using tooth #22 (implant site) as an abutment was based on the substantial alveolar bone loss and poor periodontal prognosis of the adjacent teeth (#21, missing; #23, stable). Following standard prosthetic protocols, a cantilever restoration or single-unit crown was considered less favorable given the compromised periodontal support and esthetic considerations [18].

**Figure 11:**
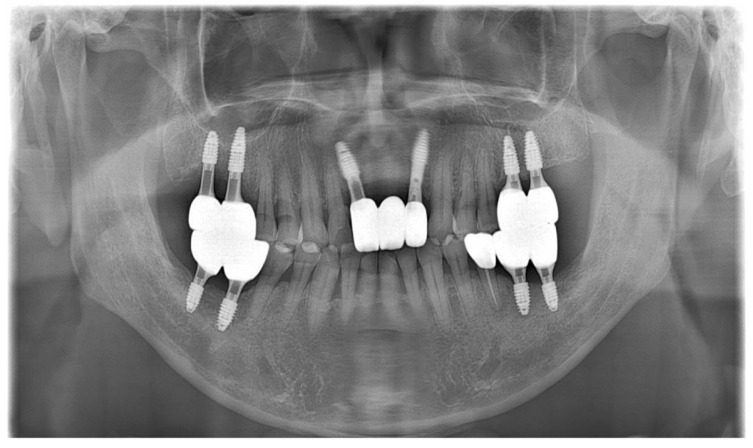
Panoramic radiograph after 2 years.

As part of the patient’s ongoing maintenance therapy, follow-up examinations were conducted every six months with routine dental plaque control. When required, additional periodontal treatment was provided during subsequent visits. Annual panoramic radiographs were taken to monitor the long-term prognosis of the implant. At the two-year follow-up, the implant prosthesis remained well maintained and functionally stable within the oral cavity (Figure 11).

### 2.2. Case Report 2

**Figure 12:**
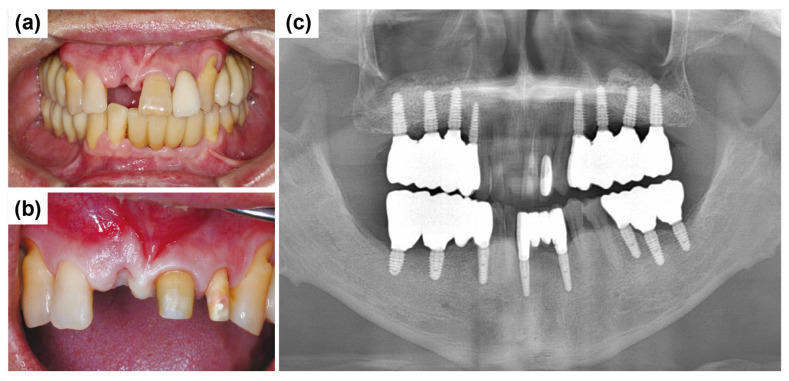
Preoperative clinical and radiographic evaluation. (**a**) Missing tooth #11 at the initial visit. (**b**) Preparation of teeth #21 and #22 for fabricating the new restoration. (**c**) Initial panoramic radiograph shows overall periodontal condition and alveolar bone status.

A 50-year-old male patient reported to the Mir Dental Hospital at Daegu without tooth #11. The patient stated that tooth #11 was lost to periodontal disease approximately five months before his visit. He was a non-smoker and had no significant systemic conditions. Intraoral examination confirmed the extraction site of tooth #11 (Figure 12a). Clinical inspection revealed severe alveolar bone loss around the apex of the missing tooth #11. The patient expressed concerns regarding the color and morphology of teeth #21 and #22, requesting esthetic improvements. Consequently, the treatment plan was expanded to include new prosthetic restorations for teeth #21 and #22 (Figure 12b). Figure 12c shows the initial panoramic radiograph, illustrating the patient’s overall periodontal condition and alveolar bone status.

After intraoral and radiographic examinations, the patient underwent scaling before implant placement. The patient was prescribed an antibiotic prophylaxis regimen consisting of 500 mg of amoxicillin sodium (Augmentin; Ilsung Pharmaceutical, Seoul, Republic of Korea) three times daily, starting one day before surgery and continuing for seven days postoperatively. The surgery was performed under local anesthesia using lignocaine 2% with noradrenaline.

**Figure 13:**
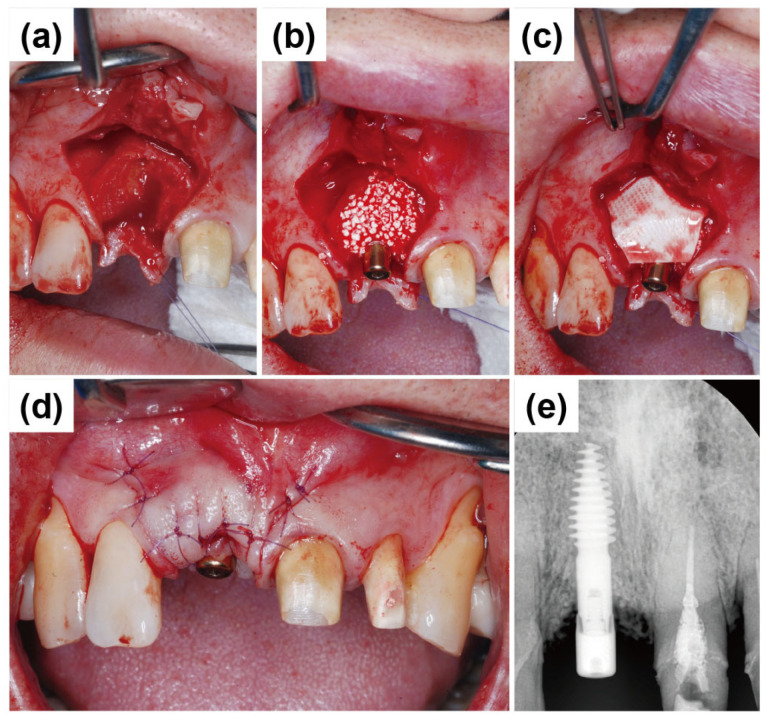
Intraoperative images and postoperative periapical radiograph. (**a**) Elevation of a full-thickness mucoperiosteal flap at the implant placement site (#11), showing a severe buccal alveolar bone defect. (**b**) Placement of a 4.1 × 10.0 mm ARi^®^ with synthetic bone graft substitute applied to augment labial bone volume and structural support. (**c**) Coverage of the grafted area with a Lyoplant collagen membrane to stabilize the graft material and prevent particle dispersion. (**d**) Primary flap closure achieved with interrupted 4-0 nylon sutures to ensure optimal healing and soft tissue adaptation. (**e**) Immediate postoperative periapical radiograph demonstrating appropriate implant positioning and initial stability.

Figure 13a illustrates the elevation of a full-thickness mucoperiosteal flap for implant placement at the site of #11. Vertical releasing incisions were made at the line angles of the adjacent teeth, along with a crevicular incision, while preserving the interdental papilla. A severe socket wall defect was observed in the buccal alveolar bone at the extraction site of #11. Additional debridement was performed using hand instruments to remove granulation tissue and prepare the site for implant placement. Due to substantial labial bone loss, an ARi^®^ Implant was selected to achieve primary stability through basal bone engagement. A 4.1 × 10.0 mm implant (ARi^®^ Implant, MEGAGEN Implant Co., Ltd., Daegu, Republic of Korea) was placed at the site (#11) with the proper initial fixation torque of 40 N/cm^2^. Figure 13b shows labial bone augmentation using a synthetic bone graft substitute to enhance ridge volume and structural support. A Lyoplant collagen membrane (Lyoplant, Aesculap AG, Tuttlingen, Germany) was carefully positioned over the grafted area to prevent graft particle displacement and promote stabilization (Figure 13c). After thorough saline irrigation, the mucoperiosteal flap was repositioned and primarily closed using interrupted 4-0 nylon sutures (Figure 13d). Figure 13e presents a postoperative radiograph of the anterior region, confirming the final positioning of the implant. Favorable healing was expected, supporting long-term stability and successful osseointegration. After a sufficient healing period of 3 months, a decision to initiate the prosthetic treatment procedure was made.

**Figure 14:**
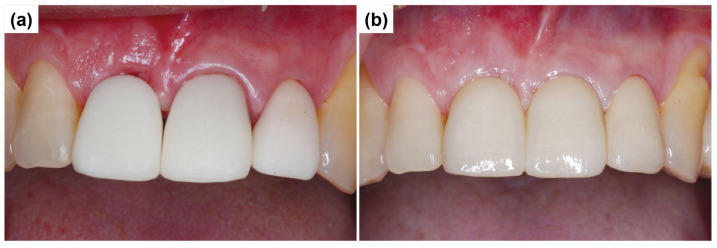
Connection of customized abutment and provisional prosthesis placement. (**a**) Customized abutment connection. (**b**) Provisional prosthesis.

Three months after the surgery, the gingival healing status was evaluated, and customized abutments were fabricated and connected to the implants (Figure 14a). Subsequently, a PMMA (polymethylmethacrylate) temporary crown was fabricated, and the provisional prosthesis was attached to the customized abutments using temporary cement (Figure 14b).

**Figure 15:**
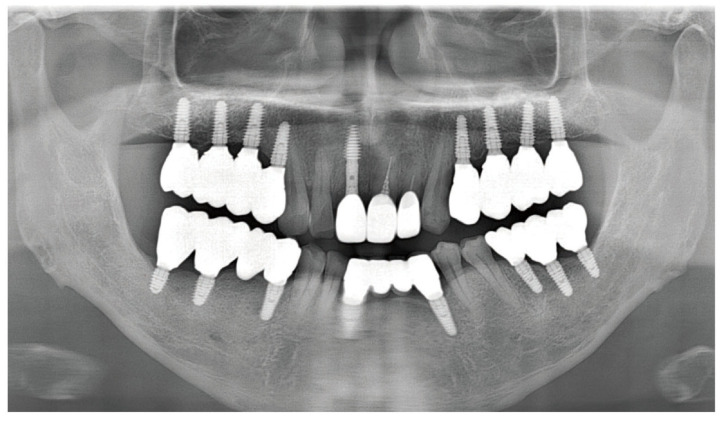
Panoramic radiograph after 2 years.

As ongoing maintenance, six-monthly follow-ups with dental plaque control were conducted. Additional periodontal treatment was provided as required. Annual panoramic radiographs were obtained to monitor implant prognosis. At the two-year follow-up, the implant prosthesis remained stable (Figure 15).

## 3. Results and Discussion

This case report evaluates the clinical applicability of the ARi^®^ Implant System for rehabilitating severely resorbed anterior alveolar ridges. Implant placement in the anterior region remains challenging due to stringent esthetic demands and insufficient bone volume, often necessitating extensive bone augmentation procedures such as guided bone regeneration (GBR) [19,20]. Previous studies by Buser et al. [20] and Simion et al. [21] highlighted prolonged healing periods and increased patient morbidity associated with complex regenerative surgeries under compromised alveolar ridge conditions.

Basal cortical implants have been proposed to address these issues by anchoring implants into dense, resorption-resistant basal cortical bone rather than relying on cancellous alveolar bone. The cortical bone, known for its high mechanical resilience, theoretically provides stable anchorage even in severely resorbed ridges [22,23]. In this study, the ARi^®^ Implant System successfully achieved primary stability through basal cortical engagement, facilitating functional restoration in patients with significant horizontal and vertical ridge deficiencies. However, it is important to emphasize that the extent of necessary bone grafting and regeneration primarily depends on the degree of alveolar ridge resorption rather than on implant design alone. Consequently, although basal cortical implants such as the ARi^®^ system offer good primary stability, the need for adjunctive bone grafting procedures, particularly in the anterior esthetic zone, is not eliminated.

Both cases required additional bone grafting techniques and membrane coverage to adequately restore ridge morphology and support soft tissue outcomes. Specifically, in Case 1, the vascularized interpositional periosteal (VIP) flap was combined with biphasic calcium phosphate (BCP) grafting and collagen membrane placement, enhancing both ridge contour and soft tissue stability. In Case 2, synthetic bone grafting and the collagen membrane were sufficient to address the ridge deficiencies without flap advancement. These adjunctive approaches contributed significantly to the stability of peri-implant tissues and improved esthetic outcomes. Nonetheless, the management of papillary recession remained clinically challenging in the esthetically sensitive anterior regions. Additionally, the apical implant placement observed in Case 2 resulted in an extended transmucosal pathway, potentially complicating long-term oral hygiene maintenance. This underscores the importance of individualized maintenance protocols and stringent plaque control to prevent peri-implant complications.

Biomechanical studies have documented the advantages of basal implants in efficiently distributing occlusal forces, thus potentially reducing crestal bone stress and marginal bone loss compared to traditional endosteal implants [24,25]. Finite element analyses (FEAs) further support the biomechanical benefits of cortical anchorage under functional loading conditions [26,27]. The ARi^®^ Implant’s macro-design, featuring deep threads tailored for cortical engagement, aligns well with these biomechanical principles. Nevertheless, these biomechanical advantages must be validated through long-term clinical trials to fully ascertain their relevance to clinical outcomes.

Importantly, while the ARi^®^ Implant System was specifically highlighted in this report for anterior ridge reconstruction, it is worth noting that basal cortical implants could also have clinical utility in posterior regions where short implants have demonstrated effectiveness. Indeed, randomized controlled trials (RCTs) have indicated favorable outcomes with short implants in posterior zones, reducing the need for complex bone regeneration surgeries and achieving reliable stability and long-term success [16,17]. This evidence suggests potential to extend the clinical application of basal cortical implant systems, including the ARi^®^ system, beyond anterior esthetic reconstructions; future comparative studies involving posterior sites would be beneficial to validate these conclusions.

This study’s findings should be interpreted cautiously due to its inherent limitations, such as the limited sample size and relatively short follow-up period, which restrict generalizability. A further limitation of this study is the lack of detailed quantitative comparisons regarding alveolar bone thickness. Conventionally, successful implant placement in the anterior region typically requires an alveolar bone width of at least 5–7 mm. In compromised cases, additional bone augmentation is often necessary to meet these criteria. Although the ARi^®^ Implant System, which was designed for basal cortical anchorage, appears beneficial in narrower ridges, the current case report did not systematically measure or provide specific quantitative bone thickness values. Consequently, it was not possible to directly compare and quantitatively validate the advantages of this implant system over conventional implants regarding bone thickness requirements. Future prospective studies should include systematic quantitative bone measurements, such as precise ridge width evaluations from CBCT, to clarify these comparative aspects. Further large-scale clinical trials with extended follow-up durations and detailed biomechanical evaluations, such as FEA, are required to comprehensively establish the clinical efficacy and long-term stability of the ARi^®^ Implant System.

## 4. Conclusions

The ARi^®^ Implant System provides a promising solution for restoring severely resorbed anterior alveolar ridges through effective basal cortical anchorage. While this approach may reduce extensive bone augmentation, it does not eliminate the need for bone grafting procedures in highly compromised anterior sites. The combination of basal anchorage, VIP flap, and BCP grafting demonstrated satisfactory esthetic and functional outcomes, which were maintained at the two-year follow-up. Given the limited number of cases and follow-up duration, further research involving larger samples and comprehensive biomechanical assessments is necessary to confirm the long-term efficacy and limitations of this implant system.

## Data Availability

No new data were created or analyzed in this study.
